# Supplementary material for: Comprehensive evaluation of high-oleic rapeseed (Brassica napus) based on quality, resistance, and yield traits: A new method for rapid identification of high-oleic acid rapeseed germplasm
Source: PLoS One. 2022 Aug 18;17(8):e0272798. doi: 10.1371/journal.pone.0272798 (PMC9387780; doi:10.1371/journal.pone.0272798)
Supplement: Attachment — Submitted filename: Manuscript JA edits.docx [file pone.0272798.s002.docx]

**Comprehensive Evaluation of of High-Oleic Rapeseed (*Brassica napus*) based on Quality, Resistance, and Yield Traits: A new method for rapid identification of high-oleic acid rapeseed germplasm**

**Tao Chang^1,2^, Junjie Wu^1,2^, XuePeng Wu^1,2^, Mingyao Yao^1,2^, Dongfang Zhao^1,2^, Chunyun Guan^1,2,3^, Mei Guan^1,2,3*^**

1 Hunan Branch of National Oilseed Crops Improvement Center, Changsha 410128, China;

2 College of Agriculture, Hunan Agricultural University, Changsha 410128, China

3 Southern Regional Collaborative Innovation Center for Grain and Oil Crops in China, Changsha 410128, China

* Correspondence: gm7142005@hunau.edu.cn

**Abstract:** To scientifically evaluate and utilize high-oleic acid rape germplasm resources and cultivate new varieties suitable for planting in the Hunan Province, 30 local high-oleic acid rape germplasms from Hunan were used as materials. The 12 personality indices of quality, yield, and resistance were comprehensively evaluated by variability, correlation, principal component, and cluster analyses. The results of variability showed that except for oleic acid, the lowest coefficient of variation was oil content, which was 0.06. Correlation analysis showed that oil content was positively correlated with main traits such as yield per plant and oleic acid, which could be used in the early screening of high-oleic rape germplasm. The results of principal component analysis showed that the 12 personality indicators were integrated into four principal components, and the cumulative contribution rate was 62.487%. The value of comprehensive coefficient ‘F’ was positively correlated with the first, second, and fourth principal components and negatively correlated with the third principal component. Cluster analysis showed that 30 high-oleic rape germplasms could be divided into four categories consisting of 9 (30%), 6 (20%), 7 (23%), and 8 (27%) high-oleic rape germplasms, each with the characteristics of "high disease resistance", "high yield", "high protein", and "more stability". This study not only provides a reference basis for high-oleic rape breeding but also provides a theoretical basis for their early screening.

**Keywords:** high-oleic acid rape; quality traits; yield traits; resistance traits; germplasm resource

**1. Introduction**

Oilseed rape (*Brassica napus* L.) is the largest oil crop in China. The output of rapeseed oil accounts for more than 50% of the output of domestic oil crops, and plays an important role in maintaining the national edible oil supply security strategy [1]. With the improvement in living standards, people's demand for enhanced edible vegetable oil quality is increasing year by year. In the past, linoleic and linolenic acid were considered the essential fatty acids for the human body; hence, they have attracted much attention [2-3]. However, recent studies have shown that oleic acid is the more important essential fatty acid [4]. High-oleic rapeseed oil not only does not smoke when heated to a higher temperature during cooking [5] but also protects cardiovascular health [6], which is favored by the majority of consumers.

~~Since 2020, Guan Chunyun, an academician in~~ The research teams at the Chinese Academy of Engineering and Hunan Agricultural University, have formulated the national standard for high-oleic rapeseed (NY/T 3786-2020), and its production has officially become an industrialized system. At present, the strategic goal for rape production is to further improve the quality and healthcare properties while ensuring or maintaining a high yield [7-8]. However, the production of high-oleic rape has been affected by problems such as reduced yield, prolonged growth period, low economic benefit, and limited promotion, which are affected by the breeding process [9]. Previous relevant studies were limited to unilateral character improvement [10-12] and failed to consider scientific and effective means to associate oleic acid with other characteristics, resulting in the slow progress of variety improvement and becoming a bottleneck in the development of the rape industry.

Crop quality, yield, and resistance are quantitative traits controlled by multiple genes [13-15]. Due to the correlation between different forms, it is difficult to analyze them comprehensively. Multivariate statistical analyses, including variation, correlation, principal component, and cluster analyses, are widely used in the comprehensive evaluation and analyses of oilseed rape traits [16-17]. Principal component analysis can combine multiple interrelated quantitative trait indices into a few independent principal components by reducing the dimension [18-19]. Cluster analysis can effectively classify all materials and preliminarily judge the genetic relationship between different varieties [20-21].

In recent years, researchers have carried out many studies related to the comprehensive evaluation of rape varieties, providing a reference basis for breeding rape varieties [22-24]. However, to the best of our knowledge, there are no relevant studies available on high-oleic rape. At present, there are no reports on some characteristics of high-oleic rape or their evaluation globally. There are few reports on the comprehensive evaluation of yield and agronomic traits of high-oleic acid rape using multivariate statistical methods. The variation, correlation, principal component analysis, and cluster analysis of the quality, yield, and resistance traits of high-oleic rape can provide a scientific and effective theoretical basis for breeding high-oleic rape.

Germplasm resources are an important basis for biological research and genetic breeding of oilseed rape [25]., Varieties which are developed and evaluated in local conditions are often better suited to local natural or cultivation conditions than foreign varieties and have excellent traits or genes to adapt to the local environment [26]. This research in transforming ordinary rapeseed oil into high-oleic one, conducted in the Hunan Province , which is a large province producing rapeseed oil, could provide huge economic benefits and nutritional benefits by contributing to the health of 61 million people in this area. , , This study collected the natural high-oleic acid rape resources in the Hunan Province and comprehensively evaluated the quality, yield, and resistance traits. These traits which are conducive to the introduction and domestication of local varieties and the excavation and utilization of excellent trait genes to lay a foundation for the cultivation of new high-oleic acid varieties and the sustainable development of the industry.

**2. Materials and Methods**

*2.1. Overview of the Experimental Field*

The experimental site was located in the Yunyuan Base of the Hunan Agricultural University, Changsha, Hunan, China (113°070’ E, 28°180’ N), with a humid subtropical monsoon climate. During the decomposition period (September 2020 to May 2021), the cumulative rainfall was 158.3 mm, and the average temperature was 16.2°C. The experimental field implemented a “rice-oilseed” rotation.

*2.2 Test Methods*

Ten plants for each material were selected, and the whole plant was harvested to detect the quality and yield traits when rapeseeds were mature. The quality traits of the oilseed rape samples which were measured included crude protein oil, and oleic, palmitic, stearic, linoleic, linolenic, and erucic acid content. The oleic, palmitic, stearic, linoleic, linolenic, and erucic acid content were determined using gas chromatography [27]. The crude protein was determined using near-infrared analysis [28], and the oil content was measured using the soxhlet method[29]. The yield traits of samples included 1000-grain weight and yield per plant, which were conducted as described by Zhao [30]. The resistance traits of samples were detected on day 28 after the pod appearance and included incidence rates of *Sclerotinia sclerotiorum* and viral diseases, which were performed as described by Wei [31].

*2.3 Materials*

The test material consisted of 30 *Brassica napus* plants with high oleic acid content. Among them, materials 1–4 were approved varieties of *Brassica napus* with high oleic acid, respectively, named Xiangyou 708, Xiangyou 710, Xiangzayou 991, and Xiangzayou 992, while materials 5–30 were stable inbred lines of *Brassica napus* with high oleic acid. The origin of these high-oleic rapes was in Chasha, Hunan province. These materials were provided by the Hunan branch of the National Oil improvement Center.

*2.4. Date analyses*

Microsoft Excel 2003 was used for statistical analyses. SPSS 24.0 was used for cluster, correlation, and principal component analyses. The comprehensive score *F* of principal components and phenotypic traits were calculated and then combined with stepwise regression analysis to screen the evaluation indices of comprehensive characters. The boxplot of traits was drawn using SPSS 24.0. The membership function analysis, genetic diversity index, and stability evaluation of germplasm resources were conducted as described by Kumar [32].

**3. Results and Analysis**

*3.1.* *Frequency distribution and diversity of traits in high-oleic rapes*

The traits of quality, yield, and resistance are shown in Figure 1. The results showed that the oleic acid content of high-oleic rapes was evenly distributed between 75–84%.

Protein mostly showed a concentration in the range of 15–25% with a frequency of 83%. The palmitic acid content was mostly concentrated in 3–5% with a frequency of 93%. The range of stearic acid was small, showing values between 1–2%. The linoleic acid in high-oleic rape was mainly less than 21% with 86% frequency. The linolenic acid was primarily concentrated in 4–8% with a frequency of 86%. Erucic acid was not present in high-oleic rape except in some cases. The oil content of high-oleic rape was largely more than 40% but not more than 46% with 83% frequency, which is a medium oil production level. The 1000-grain weight was evenly distributed between 3–4 g and yield per plant was almost concentrated in 18–27 g with 96% frequency. The incidence rate of *Sclerotinia* was higher than that of viral diseases in high-oleic rapes, but it was lower than 25%.

**Figure 1.** Variation distribution of qualitative traits of high-oleic rape germplasms

OL: Oleic, CP: Crude protein, PA: Palmitic acid, ST: Stearic acid, LI: Linoleic acid, LO: Linolenic acid, ER: Erucic acid, OC: Oil content, GW: 1000-grain weight, YP: Yield per plant, IS: Incidence rate of sclerotinia sclerotiorum, IV: Incidence rate of viral diseases. The percentages of oil content and crude protein were obtained as their percentages in total biomass. The percentages of *Sclerotinia sclerotiorum* and viral diseases were obtained as their incidence rate in these plant materials. The same as below.

*3.2. Variation analyses of quality, yield, and resistance traits of different high-oleic rape germplasms*

The 12 traits of 30 high-oleic rapeseed showed different degrees of variation, as shown in Table 1. Under the influence of double low rapeseed breeding, the coefficient of variation of erucic acid was greater than 1, but the standard deviation was small. Except for erucic acid, the coefficients of variation of other traits were low, indicating that the high-oleic rapeseed germplasms in Changsha tended to be stable. However, the standard deviation of some traits such as oleic acid, crude protein, linoleic acid, and the incidence rate of *Sclerotinia sclerotiorum* is high. In other words, within a specific range, these traits showed diversity. The results showed that the maximum *Sclerotinia sclerotiorum* concentration in high-oleic rape was more than three times the minimum value, and the average was 16.42, with a range of 8.3-29.1.

Table 1 Variation coefficients of traits in different high-oleic rapeseed

| Trait | Min | Max | AV | SD | CV |
| --- | --- | --- | --- | --- | --- |
| Quality trait |  |  |  |  |  |
| OL/% | 75.86 | 85.21 | 80.57 | 3.00 | 0.04 |
| CP/% | 12.27 | 27.74 | 21.26 | 3.45 | 0.16 |
| PA/% | 2.75 | 5.28 | 4.21 | 0.55 | 0.13 |
| ST/% | 1.01 | 1.98 | 1.52 | 0.31 | 0.21 |
| LI/% | 10.53 | 24.08 | 17.17 | 3.69 | 0.26 |
| LO/% | 3.93 | 8.51 | 6.12 | 1.32 | 0.27 |
| ER/% | 0 | 0.51 | 0.04 | 0.10 | 2.84 |
| Yield trait |  |  |  |  |  |
| OC/% | 35.52 | 48.23 | 42.90 | 2.71 | 0.06 |
| GW/g | 3.08 | 3.95 | 3.47 | 0.26 | 0.07 |
| YP/g | 18.36 | 27.52 | 22.38 | 2.59 | 0.12 |
| Resistance trait |  |  |  |  |  |
| IS/% | 8.3 | 29.1 | 16.42 | 4.80 | 0.29 |
| IV/% | 1.1 | 2.6 | 1.71 | 0.38 | 0.22 |

Min: Minimum, MAX: Maximum, AV: Average, SD: Standard deviation, CV: Coefficient of variation; Note: The percentages of oleic, palmitic, stearic, linoleic, linolenic and erucic acid were obtained as percentages of these components in fatty acid.

*3.3. Correlation analyses of quality, yield, and resistance traits of different high-oleic rape germplasms*

The correlation analyses of quality, yield, and resistance trait indices of high-oleic rape germplasms in Figure 2 showed different degrees of correlation among the traits. The results showed that oleic acid had significantly high positive correlations with stearic acid, significant positive correlations with crude protein and linoleic acid, and significant negative correlations with erucic acid, oil content, and yield per plant. Crude protein exhibited a significantly high positive correlation with linoleic acid, a significant positive correlation with linolenic acid, and significant negative correlations with erucic acid, yield per plant, and incidence rate of viral diseases. Palmitic acid had a significantly high positive correlation with linoleic acid, significant positive correlations with yield per plant and incidence rate of viral diseases, and a significantly high negative correlation with erucic acid. Stearic acid showed significant positive correlations with linoleic acid and incidence rate of *Sclerotinia sclerotiorum*, and significant negative correlations with linolenic acid and oil content. Linoleic acid had a significant positive correlation with the incidence rate of *Sclerotinia sclerotiorum* and a significant negative correlation with erucic acid. Linolenic acid showed a significantly high positive correlation with 1000-grain weight, a significant positive correlation with oil content, and a significantly high negative correlation with the incidence rate of *Sclerotinia sclerotiorum*. Erucic acid had significant negative correlations with 1000-grain weight, yield per plant, and incidence rate of viral diseases. Oil content displayed a significantly high positive correlation with 1000-grain weight and significant negative correlations with the incidence rate of viral diseases. The 1000-grain weight exhibited significant positive correlations with the incidence rate of viral diseases and a significant negative correlation with the incidence rate of *Sclerotinia sclerotiorum.*

**Figure 2.** Correlation analyses of traits of different high-oleic rape germplasms

*3.4.* *Principal component analyses (PCA) of quality, yield, and resistance traits of different high-oleic rape germplasms*

The PCA results of quality, resistance, and yield characteristics of high-oleic rape are shown in Figure 3. The results showed that most varieties were grouped together, indicating that the data were reliable and stable (Figure 3a). The PCA data analyses showed that the eigenvalues of the first four principal components were λ ≥1. The cumulative contribution rate of the first four principal components was 62.487%, which represented 62.487% of the traits of 30 high-oleic rape germplasms, and could be evaluated by the first four principal components.

The variance contribution rate of the first principal component was 19.506%. The first principal components were mainly linolenic acid, 1000-grain weight, erucic acid, *Sclerotinia sclerotiorum* incidence rate, and oil content, and their load values were 0.666, 0.635, −0.550, −0.505, and −0.500, respectively. This composition mainly reflected linolenic acid, 1000-grain weight, erucic acid, Sclerotinia sclerotiorum incidence rate, and oil content. The variance contribution rate of the second principal component was 18.009%. The main character indices determining the two principal components were linoleic, stearic, and oleic acid, and their load values were 0.649, 0.642, and 0.597, respectively. These mainly reflected linoleic, stearic, and oleic acid. The variance contribution rate of the third principal component was 14.538%. The third principal components were mainly crude protein, yield, and virus incidence rate, and their load values were −0.700, 0.683, and 0.514, respectively. These mainly reflected crude protein, yield, and incidence rate of viral diseases. The variance contribution rate of the fourth principal component was 10.435%. The fourth principal components were mainly palmitic acid and the incidence rate of viral diseases, and their load values were 0.506 and −0.505, respectively. These mainly reflected palmitic acid and the incidence rate of viral diseases. Moreover, each trait was in a different interval (3b) in cluster analysis, which could be further used to establish a model for comprehensive evaluation.

**A**

**B**

**Figure 3.** Principal component analyses (PCA) of varieties (A) and traits (B)

*3.5. Comprehensive evaluation of quality, yield, and resistance traits of different high-oleic rape germplasms*

To eliminate the influence of data dimension in the comprehensive scoring of 30 high-oleic rape germplasms, the average value of each trait index was standardized using SPSS. According to the standardized value and load matrix, the linear equation of each principal component score was obtained and then the variance contribution rate corresponding to the four principal components was used as the weight coefficient to establish a comprehensive evaluation model for comprehensive evaluation score. The linear equation and comprehensive evaluation model of the four principal component scores were as follows:

*F*_1_=0.341*X*_1_+0.454*X*_2_+0.421*X*_3_-0.137*X*_4_+0.301*X*_5_+0.666*X*_6_-0.550*X*_7_+0.500*X*_8_-0.635*X*_9_+0.045*X*_10_-0.505*X*_11_+0.276*X*_12_

*F*_2_=0.597*X*_1_+0.192*X*_2_+0.340*X*_3_+0.642*X*_4_+0.649*X*_5_-0.303*X*_6_-0.440*X*_7_-0.492*X*_8_-0.260*X*_9_+0.055*X*_10_+0.436*X*_11_+0.171*X*_12_

*F*_3_=-0.248*X*_1_-0.700*X*_2_+0.333*X*_3_+0.070*X*_4_-0.255*X*_5_-0.277*X*_6_-0.362*X*_7_+0.112*X*_8_+0.234*X*_9_+0.683*X*_10_+0.085*X*_11_+0.514*X*_12_

*F*_4_=-0.431*X*_1_+0.205*X*_2_+0.506*X*_3_-0.272*X*_4_+0.369*X*_5_-0.093*X*_6_-0.021*X*_7_+0.226*X*_8_-0.321*X*_9_+0.365*X*_10_+0.086*X*_11_-0.505*X*_12_

*F*=(19.506*F*_1_+18.009*F*_2_+14.538*F*_3_+10.435*F*_4_)/62.487

Where, *X*_1_~*X*_12_ represent oleic acid, crude protein, and palmitic, stearic, linoleic, linolenic, and erucic acid, oil content, 1000-grain weight, yield per plant, and the incidence rate of *Sclerotinia sclerotiorum* and viral diseases, respectively.

The comprehensive score of each high-oleic acid rapeseed germplasm was calculated and ranked using the above model (Table 4). The results showed that in addition to the new varieties that have been put into production, 6 materials had a high comprehensive evaluation, of which germplasm No. 14 ranked first or can be used to register new varieties. The germplasm No. 5, No. 26, No. 15, No. 6, and No. 9 showed high comprehensive scores and could be used as reserves of high-oleic acid rape germplasm resources.

Table 2 Comprehensive scores of top ten high-oleic rape germplasms

| Germplasm | First principal component score *F*_1_ | Second principal component score *F*_2_ | Third principal component score *F*_3_ | Fourth principal component score *F*_4_ | Comprehensive score *F* | Rank |
| --- | --- | --- | --- | --- | --- | --- |
| 14 | 1211.74 | 1156.94 | -241.71 | -33.67 | 33.49 | 1 |
| 3 | 1422.10 | 851.44 | -252.58 | -11.98 | 32.15 | 2 |
| 5 | 1389.88 | 921.92 | -290.37 | -58.46 | 31.41 | 3 |
| 26 | 1291.59 | 1034.10 | -338.43 | -25.45 | 31.39 | 4 |
| 15 | 1311.58 | 955.149 | -261.51 | -44.53 | 31.37 | 5 |
| 1 | 1308.40 | 1010.03 | -302.18 | -56.34 | 31.36 | 6 |
| 6 | 1379.87 | 980.16 | -323.99 | -78.80 | 31.32 | 7 |
| 9 | 1237.47 | 1045.98 | -249.62 | -77.13 | 31.31 | 8 |
| 4 | 1440.75 | 810.20 | -254.13 | -48.04 | 31.18 | 9 |
| 12 | 1214.59 | 880.18 | -144.84 | -6.89 | 31.09 | 10 |

The scores of the four principal components were used as ordinate, and the comprehensive ranking was used as abscissa. The trends of map were observed, and the results are shown in Figure 4. The results showed that principal components 1, 2, and 4 showed a downward trend ranking, while principal component 3 displayed an upward trend in terms of ranking. Among them, the fitting coefficient R-value of principal component 2, which was 0.3722, and ranking were the highest. The fitting coefficient R-value of principal component 3 was 0.1713, and the ranking was the lowest.

**B**

**A**

**Figure 4.** Trend of comprehensive score.

**C**

**D**

A: First principal component score; B: Second principal component score; C: Third principal component score; and D: Fourth principal component score. The X-axis is the ranking, and the Y-axis is each principal component.

*3.6. Cluster analyses of quality, yield, and resistance traits of different high-oleic rape germplasms*

Cluster analyses were carried out after the normalization of 12 personality traits. The results are shown in Figure 4. The results showed that 30 high-oleic rape germplasms were divided into four categories, and one group was divided into two subclasses.

**Figure 5.** Hierarchical cluster analyses of traits of different high-oleic rape germplasms

The characters of the four groups were calculated, and the results of each character are shown in Table 3. The results showed that the first category included 9 high-oleic rape germplasms, accounting for 30% of the total, which included germplasm Nos. 10, 11, 15, 19, 20, 23, 27, 28, and 29, respectively. The erucic acid content of this group was high but was still at the "double low" level, and the incidence index of this group was low. The second category included 6 high-oleic rape germplasms, accounting for 20% of the total, which included germplasm Nos. 12, 16, 18, 22, 25, and 30, respectively. The yield of the plant was higher than that of the other groups, but the incidence rate of *Sclerotium* disease increased. The third category included 7 high-oleic rape germplasms, accounting for 23% of the total. It was divided into two subclasses: subclass A included germplasm Nos. 1, 7, and 17, while subclass B consisted of germplasm Nos. 2, 3, 4, and 5. The oil content and crude protein content of this group were high. The four varieties that have been put into production belonged to this group. The fourth category included 8 high-oleic rape germplasms, which included germplasm Nos. 6, 8, 9, 13, 14, 21, 24, and 26, respectively, accounting for 27% of the total. Oleic acid content of this group was higher than that of other groups, but the incidence rate of viral disease increased.

Table 3 Significance analyses of the differences in the average values of the four groups

| Group | OL | CP | PA | ST | LI | LO | ER | OC | GW | YP | IS | IV |
| --- | --- | --- | --- | --- | --- | --- | --- | --- | --- | --- | --- | --- |
| I | 80.97  ab | 21.52b | 3.80a | 1.63a | 15.05b | 5.92  ab | 0.10a | 42.72  ab | 3.35a | 21.11  b | 15.95b | 1.42  b |
| II | 76.83c | 18.35c | 4.39a | 1.36a | 15.84b | 5.25  b | 0.02b | 42.56  ab | 3.35a | 24.64  a | 18.95  a | 1.76  ab |
| III | 80.43b | 23.54a | 4.30a | 1.23b | 18.97a | 6.90  a | 0.00b | 44.66  a | 3.74a | 21.78  b | 13.14  c | 1.70  ab |
| IV | 83.06a | 21.17b | 4.44a | 1.78a | 18.99a | 6.34ab | 0.00b | 41.82  b | 3.46a | 22.65  ab | 17.93  a | 2.03a |

Different lowercase letters in each column indicate significant differences among treatments (p<0.05).

**4. Discussion**

*4.1. Evaluation and utilization of high-oleic rape resources*

*Brassica napus* breeding materials maintain higher genetic diversity due to natural and artificial selection [33]. Previous studies on breeding materials mostly focused on yield-related traits, which made the materials with high-quality potential not be fully utilized [34-35]. In recent years, with changes in the orientation of oilseed rape breeding, the goal has gradually changed to high-quality and multi-purpose varieties. Re-evaluation and utilization of breeding resources is an important way to expand the genetic basis of oilseed rape breeding and make full use of the seed. In addition, the studies on the genetic diversity of quality traits of breeding materials mostly focus on specific types, which is related to the clustering results showing specific groups. The 30 high-oleic rapeseed materials selected in this study showed multiple phenotypes such as "high oil content", "high protein", and "high disease resistance". It was found that the diversity index of other characteristics except erucic acid was less than 1.00. The results showed that the overall changes in the trend of high-oleic rapeseed germplasm resources in the Hunan Province is consistent. However, the standard deviation of some traits such as oil content, protein, and *Sclerotinia* resistance were large, indicating a possibility for further improvement in high-oleic rape breeding.

*4.2. Comprehensive evaluation and index screening of phenotypic characters of high-oleic rape germplasm resources*

The quality, yield, and resistance traits of rapes are complex quantitative traits affected by their own genetic factors and external environmental conditions [36-37]. It is difficult to accurately and objectively evaluate the quality of germplasm resources using a single index. In particular, focusing on only the content of oleic acid in high-oleic rapes could result in low yield and serious disease susceptibility. Therefore, this study used multivariate statistical analysis to evaluate high-oleic rape. These methods have been applied in the comprehensive evaluation of rice [38], rape [22], cotton [39], millet [40], and peanut [41]. The evaluation results for this reserach showed that the approved rape varieties, No.1, 3, and 4 were in the top 10 of the evaluation, indicating that the data statistics were stable and reliable. However, variety No. 2 ranked 22^nd^ during our evaluation. Thus, we can speculate that the quality of this variety may have declined slightly after many years of planting because it is a conventional species, which is consistent with the "maintenance breeding" theory [42]. Therefore, continuous and uninterrupted breeding of high-oleic rape resource is not only the guarantee of maintaining rape quality but also an assurance of rape yield and resistance.

This study found that the first, second, and fourth principal components showed an upward trend, and the third principal component showed a downward trend in terms of ranking, but the fitting coefficient R-value did not show significance. The reason for this is the limitation of univariate regression simulation. The selection of multivariate or other fitting methods could improve the fitting coefficient. However, even with an increase in the coefficient, the trend cannot change. Therefore, selectively increasing the first, second, and fourth principal components or reducing the traits represented by the third principal component can improve the quality of high-oleic rape varieties. However, it should be noted that there was a saturation range for some traits during selection. For example, the content of erucic acid, the first principal component, was low in this study. An appropriate increase is conducive to the synthesis of fatty acids such as oleic acid, but after reaching a certain degree, this increase may affect the quality. This study can be taken as a breakthrough in the correlation between yield and quality of high-oleic rape, and provide a reference basis for breeding new rape varieties with high yield and improved quality.

*4.3. Correlation and application among quality, yield, and resistance traits*

There are correlations among many traits of rape, which can be used as the basis for screening specific germplasm resources. In this study, the correlation analysis of multiple trait indices of high-oleic rape showed for the first time that there was a significantly negative correlation between oil and protein content. This was consistent with the results of oil and protein concentration in rape by Delourme [43], and also in agreement with the "substrate competition" hypothesis of Chen Jinqing [44]. At the same time, this study also found a significant positive correlation between oil content and yield per plant. The reason may be that these two traits can control yield and affect each other. Previous studies have shown a significant correlation between chlorophyll content and yield in the early growth stage of rape, and the chlorophyll content can be efficiently measured by a spectrometer [45-47]. Combined with the above views, a rapid method for early and rapid identification of high-oleic rape can be constructed to promote the process of breeding and improvement of high-oleic rape worldwide.

**5. Conclusions**

In summary, the germplasm resources of high-oleic rape in the Hunan Province were relatively stable. The highest comprehensive evaluation score of materials was 14 in this study, which can be used to declare new varieties and put into actual production in the Hunan Province. However, the properties of other materials need to be further improved. Cluster analysis showed that the germplasm resources could be divided into four categories, with the characteristics of "high disease resistance", "high yield", "high protein", and "more stable" in high-oleic rape. Principal component analysis showed that in the range of "double low", appropriately increasing erucic acid content could improve the production of high-oleic rape. Correlation analysis showed that oil content and yield per plant could be used as indices for early screening of high-oleic rape.

**Funding**: This work was supported by “China Agriculture Research System of MOF and MARA” and “Hunan Agriculture Research System of DARA”.

**References**

1. Hatzig, S.V.; Nuppenau, J.N.; Snowdon R.J.; Schießl, S.V. Drought stress has transgenerational effects on seeds and seedlings in winter oilseed rape (*Brassica napus* L.). *BMC Plant Biology*. **2018**, 18, 297.

2. Zarhloul, M.K.; Stoll, C.; Lühs, W.; Alexandra, S.E., Hausmann, L., Töpfer, R.; Friedt, W. Breeding high-stearic oilseed rape (*Brassica napus*) with high- and low-erucic background using optimised promoter-gene constructs. Molecular Breeding. 2006, 18, 3, 241-251.

3. Figen M.T.; GÜL M.K.; Egesel, C.Ö. Nitrogen and fungicide applications against Erysiphe cruciferarum affect quality components of oilseed rape. Mycopathologia. 2008, 165, 1, 27-35.

4. Lee, Y.H.; Park, W.; Kim, K.S.; Jang, S.Y.; Lee J.E.; Cha,Y.L.; Moon, Y.H., Song Y.S.;Lee, K.; EMS-induced mutation of an endoplasmic reticulum oleate desaturase gene (FAD2-2) results in elevated oleic acid content in rapeseed (*Brassica napus* L.). Euphytica. 2018, 214, 2, 28.

5. Khan, H.M.; Ali,C.H.; Iqbal, T.; Yasin,S.; Sulaiman, M.; Mahmood, H.; Raashid, M.; Pasha, M.; Mu, B. urrent scenario and potential of biodiesel production from waste cooking oil in Pakistan: An overview. Chinese Journal of Chemical Engineering. 2019, 27, 10, 2238-2250.

6. Noriega, A.K.; Tirado, A.; Méndez, C.; Marroquín, G.; Ancheyta, J.; Hydrodeoxygenation of vegetable oil in batch reactor: Experimental considerations. Chinese Journal of Chemical Engineering. 2020, 28, 6, 1670-1683.

7. Fu, W., Luo, M.C.; Chen, J.C.; Evolution process,development goal and realization path of China’s agricultural green development. Ecological Economy, 2020, 16, 04, 317-326.

8. Chang, T.; Guan, M.; Zhou, B.Q.; Peng, Z.C.; Xing, M.; Wang, X.D.; Guan, C.Y.; Progress of CRISPR/Cas9 technology in breeding of *Brassica napus*, Oil Crop Science, 2021, 6, 02, 53-57.

9. Tian, E.T.; Liang,H.L.; Wang,J.J.; Guo, J.; Cao,H.L.; Lin,S.C.; Variation and Correlation of Erucic Acid, Oleic Acid and Glucosinolate Contents in *Brassica* rapa Seeds. Asian Agricultural Research, 2018, v.10, 06, 57-60.

10. Guo, S., Yan, Y., Dan, B. Application of Molecular Marker Technologies in Stress Resistance Breeding of Rapeseed. Asian Agricultural Research, 2020, 12, 07, 36-40.

11. Yin, J.X.; Wei, C.H.; Tang, H.Q.; Dong, W.B.; Li, Z.Y. Research Progress on Application of Rape as Green Manure. Agricultural Biotechnology, 2020, 9, 2, 64-68+72.

12. Zhang, X.X.; Liu, Q.; Hong, M.Y.; Luo, L.X.; Guo, R.X.; Analysis of yield and quality characteristics of winter oilseed rape in China from 2001 to 2015: towards future breeding goals. Oil Crop Science, 2018, 3, 3, 165-175.

13. Pott, D.M.; Soria, S.D.; Osorio, S.; Vallarino, J.G.; Combining metabolomic and transcriptomic approaches to assess and improve crop quality traits. CABI Agric Biosci, 2021, 2, 1.

14. Petrova, S.N.; Andronov, E.E.; Belimov, A.A.; Beregovaya, Y.V.; Minakov, D.L.; Prokaryotic Community Structure in the Rapeseed (*Brassica napus* L.) Rhizosphere Depending on Addition of 1-Aminocyclopropane-1-Carboxylate-Utilizing Bacteria. Microbiology. 2020, 89, 1, 115-121.

15. Aubertot, J.N.; West, J.S.; Bousset-Vaslin, L.; Salam, M.U.; Barbetti, M.J.; Diggle, A.J. Improved Resistance Management for Durable Disease Control: A Case Study of Phoma Stem Canker of Oilseed Rape *(Brassica napus*). European Journal of Plant Pathology. 2006, 114, 1, 91-106.

16. Alizadeh, B.; Rezaizad, A.; Mohammad, Yazdandoost.; Shiresmaeili, G.; Nasserghadimi, F.; Khademhamzeh, H.R.; Gholizadeh, A. Genotype × Environment Interactions and Simultaneous Selection for High Seed Yield and Stability in Winter Rapeseed (Brassica napus) Multi-Environment Trials. Agric Res. 2021.

17. Wesołowski, M.; Suchacz, B. Classification of rapeseed and soybean oils by use of unsupervised pattern-recognition methods and neural networks. Fresenius Journal of Analytical Chemistry. 2001, 371, 3, 323-330.

18. Szydłowska-Czerniak, A.; Karlovits, G.; Dianoczki, C.; Recseg, K. Comparison of Two Analytical Methods for Assessing Antioxidant Capacity of Rapeseed and Olive Oils. Journal of the American Oil Chemists Society. 2008, 85, 2, 141-149.

19. Gong, Q.;Lian, J.L.; Li, X.R.;Huang, Z.;Xu, A.X.;Dong J.G.;Yu, C.Y. The genetic diversity and heterotic groups of 169 Chinese semi-winter rapeseed (*Brassica napus*) cultivars and inbred lines. Molecular Breeding. 2020, 40, 4.

20. Anne-Sophie, B.; Anne, L.; Christine, B.B.; Cécile, B.; Jér?me, M.; Mathieu, R.G.; Jean-Eric, D.; Pierre, G.; Xavier, P.; Thomas, F.; Olivier, M.; Damien, D.; Florent, G.; Nathalie, N. Genetic basis of nitrogen use efficiency and yield stability across environments in winter rapeseed. Bmc Genetics. 2016, 17, 1, 131.

21. Tian, L.L.; Zeng,Y.Y.; Zheng, X.Q.; Chiu, Y.; Liu, T. Detection of Peanut Oil Adulteration Mixed with Rapeseed Oil Using Gas Chromatography and Gas Chromatography–Ion Mobility Spectrometry. Food Analytical Methods. 2019, 12, 10, 2282-2292.

22. Chen, B.Y.; Xu, K.; Li, J.; Li, F.; Qiao, J.W.; Li, H.; Gao, G.Z.; Yan, G.X.; Wu, X.M. Evaluation of yield and agronomic traits and their genetic variation in 488 global collections of *Brassica napus* L. Genetic Resources and Crop Evolution. 2014, 61,5, 979-999.

23. Hu, Q.; Hansen, L.; Laursen, J.; Dixelius, C.; Andersen, S. Intergeneric hybrids between *Brassica* *napus* and Orychophragmus violaceus containing traits of agronomic importance for oilseed rape breeding. Theor Appl Genet. 2020, 1, 105, 834-840.

24. Rajcan, I.; Kasha, K.J.; Kott, L.S.; Beversdorf, W.D. Evaluation of cytoplasmic effects on agronomic and seed quality traits in two doubled haploid populations of *Brassica napus* L. Euphytica. 2002, 123, 3, 401-409.

25. Obermeier, C.; Mason, A.S.; Meiners, T.; Petschenka, G.; Rostás, M.; Will, T.; Wittkop, B.; Austel, N.; Perspectives for integrated insect pest protection in oilseed rape breeding. Theor Appl Genet. 2022.

26. Jaradat, A.A. Integrating plant ontogeny and structure in *Brassica napus* L. I. Forward phenomics. Euphytica. 2018, 214, 8, 141.

27. Pham, A.T., Shannon, J.G., Bilyeu, K.D. Combinations of mutant FAD_2_ and FAD_3_ genes to produce high oleic acid and low linolenic acid soybean oil. Theoretical & Applied Genetics, 2012, 125, 3, 503-515.

28. Velasco, L.; Christian, M. Nondestructive assessment of protein content in single seeds of rapeseed (*Brassica napus* L.) by near-infrared reflectance spectroscopy. Euphytica. 2002, 123, 1, 89-93.

29. Yang, Mei.; Huang, F.H.; Liu, C.S.; Zheng, C.; Zhou, Q.; Wang, H. Influence of Microwave Treatment of Rapeseed on Minor Components Content and Oxidative Stability of Oil. Food & Bioprocess Technology, 2013, 6, 11, 3206-3216.

30. Zhao, J.Y.; Becker, H.C.; Zhang, D.Q.; Zhang, Y.F.; Ecke, W.G. Conditional QTL mapping of oil content in rapeseed with respect to protein content and traits related to plant development and grain yield. Theoretical & Applied Genetics. 2006, 113, 1, 33-38.

31. Wei, D.Y.; Mei, J.Q.; Fu, Y.; Disi, J.O.; Li J.; Qian, W. Quantitative trait loci analyses for resistance to Sclerotinia sclerotiorum and flowering time in *Brassica napus*. Molecular Breeding. 2014, 34, 4, 1797-1804.

32. Kumar, P.; Nimbal, S.; Budhlakoti, N.; Singh, V.; Sangwan, RS. Genetic diversity and population structure analysis for morphological traits in upland cotton (*Gossypium hirsutum* L.). Journal of Applied Genetics. 2022, 63, 1, 87-101.

33. Li, Z.Y.; Wang, Y.P. Cytogenetics and germplasm enrichment in *Brassica* allopolyploids in China. Journal of Integrative Agriculture, 2017, 16, 12, 2698-2708.

34. Wolko, J.; Dobrzycka, A.; Bocianowski, H.; Bartkowiak-Broda, I. Estimation of heterosis for yield-related traits for single cross and three-way cross hybrids of oilseed rape (*Brassica napus* L.). Euphytica, 2019, 215, 10, 1-17.

35. Teh, H.F.; Neoh, B.K.; Ithnin, N.; Daim, L.D.J. Review: Omics and Strategic Yield Improvement in Oil Crops. Journal of the American Oil Chemists Society, 2017, 94(10).

36. Chen, W.; Zhang, Y.; Liu, X.P.; Chen, B.Y.; Tu, J.X.; Fu, T.D. Detection of QTL for six yield-related traits in oilseed rape (*Brassica napus*) using DH and immortalized F2 populations. Theoretical & Applied Genetics. 2007, 115, 6, 849-858.

37. Liu, R.; Ding, L.N.; Li, M.; Cao, W.; Wang, Y.K.; Wang, W.H.; Yu, Y.K.; Wang, Z.; Zhu, K.M.; Tan, X.L. Characterization of a Rapeseed Anthocyanin-More Mutant with Enhanced Resistance to Sclerotinia sclerotiorum. Journal of Plant Growth Regulation. 2020, 39, 1, 703-716.

38. Liu, S; Zhao, H.F.; Wu, K.N.; Zhang, Z.; Hou, Y.X.; Chen, T.Y.; Jin, Q. Evaluation of heavy metal distribution characteristics of agricultural soil-rice system in a high geological background area according to the influence index of comprehensive quality (IICQ). Environmental science and pollution research international, 2020, 27, 17, 20920-20933.

39. Zhou, J.F.; Xu, B.G. Evaluating cotton length uniformity through comprehensive length attributes measured by dual-beard fibrography. Cellulose, 2020, 27, 13, 7861-7871.

40. Yue, H.; Deng, P.C.; Liu, S.Y.; Wang, M.; Song, W.N.; Nie, X.J. Selection and evaluation of reference genes for quantitative gene expression analysis in broomcorn millet (*Panicum miliaceum* L.). Journal of Plant Biology. 2016, 59, 1, 435-443.

41. Yang, Q.Q.; Kim, G.; Farha, A.K.; Luo, Q.; Corke, H. Phenolic profile, antioxidant and antiproliferative activities of diverse peanut cultivars. Journal of Food Measurement and Characterization. Journal of Food Measurement and Characterization. 2020, 14, 3, 2361-2369.

42. Yu, S.B.; Xu, W.J.; Vijayakumar, C. H.M.; Ali, J.; Fu, B.Y.; Xu, J.L.; Jiang, Y.Z.; Marghirang, R.; Domingo, J.; Aquino, C.; Virmani S.S.; Li Z.K. Molecular diversity and multilocus organization of the parental lines used in the International Rice Molecular Breeding Program. Theoretical & Applied Genetics, 2003, 108, 1, 131-140.

43. Delourme, R.; Falentin, C.; Huteau, V.; Clouet, V.; Horvais, R.; Gandon, B.; Specel, S.; Hanneton, L.; Dheu, J.E.; Deschamps, M. Genetic control of oil content in oilseed rape (*Brassica napus* L.). Theoretical and Applied Genetics, 2006, 113, 7, 1331-1345.

44. Wu, Q.K.; Cao, Y.Y.; Chen, C.; Gao, Z.Z.; Yu, F.Y.; Guy, R.D. Transcriptome analysis of metabolic pathways associated with oil accumulation in developing seed kernels of Styrax tonkinensis, a woody biodiesel species. BMC Plant Biol. 2020, 20, 121.

45. Siedliska, A.; Baranowski, P.; Pastuszka-Woźniak, O.; Zubik, M.; Identification of plant leaf phosphorus content at different growth stages based on hyperspectral reflectance. BMC Plant Biol. 2021, 21, 28.

46. Daun, J.K. A rapid procedure for the determination of chlorophyll in rapeseed by reflectance spectroscopy. Journal of the American Oil Chemists' Society. 1976, 53, 12, 767-770.

47. Tkachuk, R.; Mellish, V.J.; Daun, J.K.; Macri, L.J.; Determination of chlorophyll in ground rapeseed using a modified near infrared reflectance spectrophotometer. Journal of the American Oil Chemists' Society. 1988, 65, 3, 381-385.
